# Anemia Secondary to Abnormal Uterine Bleeding Requiring Blood Transfusion in the Reproductive Age Group—A Retrospective Study

**DOI:** 10.3390/life16050855

**Published:** 2026-05-21

**Authors:** Asha Santhosh, Sunita Jesrani, Jumana Al Mahruki, Tahnai Al Badi, Maryam Al Shukri, Vaidyanathan Gowri, Sachin Jose

**Affiliations:** 1 Hinchingbrooke Hospital, Cambridgeshire PE29 6NT, UK; pranjal07ochani@gmail.com; 2 Department of Obst Gyne, University Medical City, Sultan Qaboos University Hospital, Muscat 123, Oman; sjesrani@squ.edu.om (S.J.); mnalshukri@gmail.com (M.A.S.); 3 Oman Medical Specialty Board, Muscat 132, Oman; r23105@resident.omsb.org (J.A.M.); r25125@resident.omsb.org (T.A.B.);; 4 Department of Obst Gyne, College of Medicine and Health Sciences, Sultan Qaboos University, Muscat 123, Oman

**Keywords:** abnormal uterine bleeding, heavy menstrual bleeding, anemia, blood transfusion, Oman

## Abstract

Background: Women with abnormal uterine bleeding (AUB) is reported in 10–30% of women and is a significant cause of iron deficiency anemia (IDA), with long-term effects on women’s quality of life. This retrospective study looked at the underlying causes of AUB and the contributory factors that required blood transfusions. Objectives: This study aimed to identify the treatable causes of AUB in patients who needed blood transfusion and parenteral iron in the reproductive age group and provide appropriate treatment to these underlying pathologies. Methods: A retrospective study was conducted in reproductive age group women with anemia due to AUB over a period of 10 years from January 2013 to December 2022. All women with significant uterine bleeding, who required blood transfusion were included in this study. Women with pregnancy, and hemolytic anemia, were excluded from the study. Results: During the study period 266 women needed blood transfusion for AUB. The mean age was 37.66 ± 11.4 years, mean parity 3, mean body mass index (BMI): 29.4 ± 8.9 kg/m^2^, and most reported regular cycles. The most common etiology of AUB was uterine fibroids in 37.9% followed by ovulatory dysfunction in 15%. The mean hemoglobin was at 5.7 ± 1.14 gm/L, mean ferritin was 11.01 ± 21.88 ng/mL, mean number of blood transfusion was 2.83 ± 1.2 at first presentation and about 24% needed further transfusions. About 70% of them preferred oral hormonal treatment. Surgical management was required in about 35% of patients. Conclusion: The main cause of AUB was leiomyoma and anovulation. The morbidity of blood transfusion was easily avoidable in these women.

## 1. Introduction

Normal menstrual cycles are 24–38 days with an average blood loss of about 30–40 mL, requiring the use of 3–6 pads or tampons per day. Abnormal uterine bleeding (AUB) is defined as bleeding from the uterus that is abnormal in duration, volume, frequency, and/or regularity and has been present for the previous six months in most reproductive age women [1,2]. Heavy menstrual bleeding is defined as bleeding lasting more than 7 days or blood loss more than 80 mL per cycle [1].

Women with AUB are commonly encountered with moderate to severe iron deficiency anemia (IDA), requiring blood transfusion. AUB is reported in 10–30% of reproductive age women [3]. The principal goals for the management of AUB are to stop the bleeding, to correct anemia, and to maintain normal menstrual cycles. The method of treatment chosen mainly depends on the severity and cause of bleeding and future fertility prospects. If AUB is mild to moderate and negatively affects the quality of life, non-steroidal anti-inflammatory drugs (NSAIDs), tranexamic acid and/or hormonal therapy is prescribed with a follow-up in 3–6 months [4].

Patients with moderate to severe anemia often present to an acute care setting for initial assessment and evaluation. Red blood cell transfusion is usually necessary if the patient becomes significantly symptomatic or hemodynamically unstable. Potentially blood transfusion increases the risk of transfusion reactions, blood-bore infection, and alloantibody formation. Parenteral intravenous iron therapy is infrequently used despite evidence of safety and superiority to oral iron therapy in other acute care settings [5].

The International Federation of Gynecology and Obstetrics (FIGO 1) recommends the use of the term AUB to describe any aberration of menstrual volume, regulation, duration and/or frequency in a woman who is not pregnant [6]. FIGO 2 defines the etiology of AUB using the PALM-COEIN classification Polyp, Adenomyosis, Leiomyoma, Malignancy, Hyperplasia (structural causes); Coagulopathy, Ovulatory dysfunction, Endometrial, Iatrogenic and Not yet classified (non-structural causes) [7].

The focus of an initial evaluation of a patient with heavy menstrual bleeding (HMB) is to determine if the bleeding is causing hemodynamic instability, although careful history, physical examination, laboratory testing and radiologic imaging are needed to find out the cause of AUB. There is a previous study by Yaqoubi et al. from Oman about blood transfusion practices, which describes both obstetrics (pregnant) and gynecology patients. Their study included early pregnancy bleeding and about 95 women with abnormal uterine bleeding with Hb < 7 gm/L [8]. Our study included a larger sample size and focused exclusively on non-pregnant women.

## 2. Methods

### 2.1. Study Design and Setting

This was a retrospective descriptive cohort study of all women with anemia resulting from abnormal uterine bleeding that have been admitted to the Sultan Qaboos University City Hospital, Muscat, Sultanate of Oman, over 10 the period of years from January 2013 to December 2022. All females from menarche till menopause with significant uterine bleeding, with or without hemodynamic instability, who required blood transfusion were included in this study. Women with pregnancy, hemoglobinopathies, and other causes of hemolysis or nutritional anemia were excluded from the study. The data was collected from the hospital electronic patient record. The different causes of abnormal uterine bleeding according to PALM-COEIN, FIGO classification were collected. For women who underwent endometrial biopsy and surgical intervention, histopathology was included and studied in addition. STROBE check list, supplement Table S1.

Patients’ consent was not required as this was retrospective study. However, ethical approval was obtained from the Medical and Research Ethics Committee of the College of Medicine and Health Sciences, Sultan Qaboos University. (MREC # 2938, Ref no SQU_EC/014/2023).

### 2.2. Sample Size

The sample was based on the duration of the study, 10 years, and no formal sample size calculation was done.

## 3. Material

Women included are those presented to the hospital with abnormal uterine bleeding requiring blood transfusion, followed by further management of anemia and the cause of the AUB. We recorded information on ethnicity, age, parity, body mass index (BMI in kg/m^2^), comorbidities, menstrual history and severity of symptoms, vital signs at presentation, abdominal and pelvic examination. Ultrasound findings, hemoglobin and serum ferritin levels, were also recorded. Anemia was classified as mild (Hb 10–11 m/dL), moderate (Hb 7–9.9 gm/L), severe (4–6.9 gm/L) or very severe (Hb < 4 gm/L) [9]. Blood transfusion and parenteral intravenous or oral iron were used to correct the anemia. Different modalities of treatment like non-hormonal, hormonal, surgical treatment were also collected from the records. Women had surgical treatment after correction of anemia and were followed up three months after transfusion or surgical management. We evaluated the histopathological reports of the women who needed surgical intervention, to reach a final diagnosis. Furthermore, we assessed hemoglobin at a follow-up visit and compliance to the treatment, for those who needed only medical treatment.

Hemodynamic instability was prespecified as systolic blood pressure < 90 mm Hg [10]. Iron deficiency anemia (IDA) was defined by a history of acute or chronic blood loss combined with any of the following: documented diagnosis of IDA, serum ferritin < 30 ng/mL, transferrin saturation < 20%. If iron studies were not obtained during the index visit, these values were obtained at follow-up visit after blood transfusion; hemoglobin level, number of units of blood transfused during first admission, post-transfusion Hb, follow-up Hb, and need for repeated transfusion were also recorded.

## 4. Results

During the study period 266 women in reproductive age presented with AUB and with moderate to severe iron deficiency anemia who required blood transfusion. The mean age of women was 37.66 ± 11.4 years and 7.12% (*n* = 19) were less than 15 years of age. Table 1 shows the baseline characteristics of 266 participants. About 66.5% (177) were multiparous and the rest were nulliparous. In some women the hemoglobin level was 4 gm/L even after transfusion at follow-up, as they were not compliant with the treatment.

Among the 266 women who received blood transfusions, 249 presented with symptomatic anemia, while 6.3% (17/266) did not report significant anemia symptoms but had blood transfusion due to markedly low hemoglobin levels and ongoing bleeding. The majority of women presented with severe anemia, Hb of 4 to 6.9 gm/dL (91%) or 5% with very severe anemia, which is less than 4 gm/dL, necessitating blood transfusion.

The total number of blood units transfused in the initial admission per patient ranged from 1 to 7 units, with most patients (n = 145; 55%) receiving 2 units.

Post-transfusion complete blood count (CBC) showed improvement of Hb in most patients, with 88% (n = 235) classified as having moderate anemia of 7 gm/dL or above. No patients remained in the very severe category, and only two patients had persistent severe anemia due to ongoing bleeding. These two women required additional transfusion and surgical intervention in the form of therapeutic dilatation and curettage due to failure of medical therapy.

At a 3-month follow-up, hemoglobin (Hb) levels reassessment showed that: 21% (n = 58) of patients achieved normal Hb levels, 33% (n = 88) had mild anemia, and 28.2% (n = 75) had moderate anemia. A small subset (3%; n = 8) redeveloped severe to very severe anemia. Repeat transfusions were needed in 23.67% (n = 63) of patients. Regarding iron supplementation, 30% (n = 79) received intravenous iron, while 36.47% (n = 97) were managed with oral iron therapy.

The correlation between number of units of transfusion and severity of anemia is described in Table 2.

**Figure 1:**
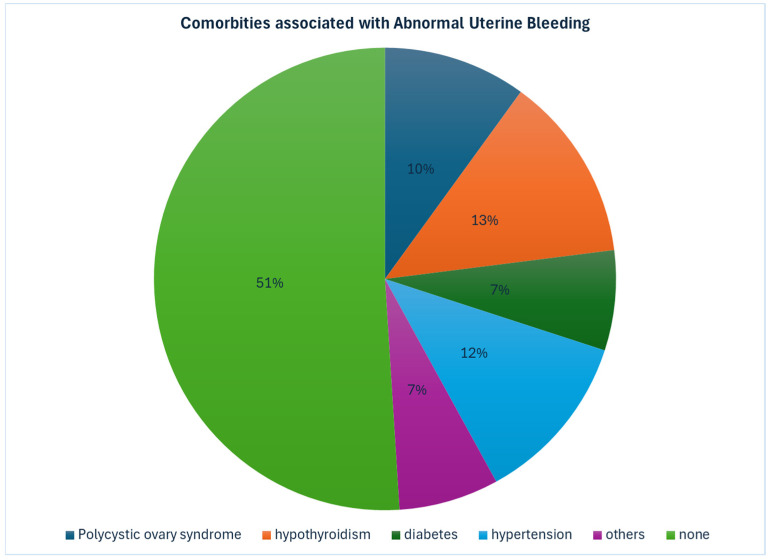
Comorbidities associated with AUB.

The mean, BMI was 29.4 ± 8.9 kg/m^2^; however, in 37 women BMI was not documented. We found that 154 out of 266 women (57.9%) presenting with AUB were either overweight or obese. In our cohort, most patients (n = 137) did not have any underlying medical conditions. Among those with comorbidities, the most common findings were hypothyroidism (n = 37), hypertension (n = 31), polycystic ovary syndrome (n = 30), and diabetes mellitus (n = 20). Other less frequent conditions included various cardiac, respiratory, and gastrointestinal disorders (Figure 1).

Most women (62.8%) reported regular menstrual cycles with HMB, suggesting that menstrual regularity did not preclude significant blood loss. Additionally, 6.3% (17/266) women reported intermenstrual bleeding, and 1.5% (4/266) experienced postcoital bleeding.

Pelvic imaging, especially transvaginal ultrasonography, revealed that the most common findings were leiomyoma and adenomyosis (47.74%), followed by thickened endometrium, polyps, and polycystic ovary syndrome. The etiologies as per FIGO classification is presented in Table 2. Thirty-two (12.04%) of our patients had combined etiologies. The most common combination is that of leiomyoma and adenomyosis as depicted in Table 3.

Histological evaluation further supported benign causes in the majority, with endometrial sampling being the main diagnostic tool. Pipelle biopsy was taken in 32.72% followed by hysteroscopy and biopsy in 22.55%. Two women needed cervical biopsy, and others did not need a biopsy. The commonest findings in Pipelle sampling and hysteroscopic biopsy were proliferative endometrium, secretory endometrium in about 23% each, followed by pseudo decidualization and inadequate 10% each.

About 42% (112) women underwent surgical management. Histopathological report is described in (Table 4). Three women underwent uterine artery embolization (1.12%).

The surgical procedures included myomectomy (laparoscopy/laparotomy/hysteroscopy), hysterectomy (laparotomy/laparoscopic/vaginal) and endometrial resection.

Medical management was the first-line approach for all patients presenting with abnormal uterine bleeding (AUB). Among these, non-hormonal therapy was the most commonly used treatment modality, administered to 81.5% of patients. Table 5 describes the hormonal and non-hormonal treatment. However, a combination of oral hormones and non-hormonal was taken in almost 60%; 10% took non-hormonal medications only. Almost 82% women took non-hormonal medications like tranexamic acid and/or mefenamic acid with other types of treatment.

Surgical intervention was performed in 42% of patients, with myomectomy (30.35%) being the most frequent procedure. Hysterectomy was performed in 15.78% of patients, typically in those with completed families or refractory symptoms. Minimally invasive procedures like hysteroscopic polypectomy (8.64%) and endometrial ablation (2.25%) were utilized based on patient suitability and histopathological findings.

Women diagnosed to have cervical cancer and endometrial cancer were referred to a cancer center for further treatment including surgical management and chemotherapy. The staging is difficult to comment as they were referred to a cancer center.

A flowchart depicts the main diagnoses and treatment modalities. (Supplement Figure S1).

## 5. Discussion

The study mainly looked at women needing blood transfusion for AUB. The majority of women in our study presented with severe (91%) or very severe (5%) anemia secondary to AUB, necessitating blood transfusion. The total number of blood units transfused per patient ranged from 1 to 7 units. Although blood transfusion is necessary, it may add to morbidity.

Of the 266 who had transfusion, some of them were asymptomatic, with very low Hb indicating very severe anemia. This spotlights the importance of both clinical assessment and objective hemoglobin evaluation in managing AUB-related anemia [11]. Transfusion of 1–2 units was typically effective in stabilizing hemoglobin and alleviating symptoms in 55% of the cases. This approach is consistent with the recommendations from the National Institute for Health and Care Excellence (NICE), which advocate individualized transfusion strategies based on both clinical presentation and Hb levels rather than numeric thresholds alone [11]. This high burden of AUB mirrors findings in prior studies that report significant anemia among women with chronic or acute uterine bleeding, particularly in regions with limited access to early gynecological care [12,13]. The majority of patients (87%) reported heavy menstrual bleeding (HMB), aligning with global data indicating that nearly half of reproductive-aged women experience HMB [14].

Comorbidities play a significant role in the presentation and management of abnormal uterine bleeding (AUB).The association between hypothyroidism and menstrual irregularities, including heavy or infrequent bleeding, is well-documented, due to anovulatory cycles as reported by Krassas et al. [15] PCOS, was another common endocrine disorder identified, which is known to cause chronic anovulation, resulting in heavy periods either regular or irregular, hypothyroidism was present in 13% and PCOS in about 10% of our women. PCOS is also associated with a risk of endometrial hyperplasia [16].

Hypertension and diabetes mellitus, both present in 12% and 7% of our patients respectively, are known to increase the risk of endometrial pathology, including hyperplasia and carcinoma [17]. Therefore, their presence warrants a more cautious diagnostic and therapeutic work up in these patients. The presence of comorbidities not only affects the choice of medical therapy but also surgical outcomes and the risk of complications. The commonest comorbidity in our women was hypothyroidism, and the next common one was polycystic ovarian syndrome (PCOS), and these diagnoses were made even prior to admission based on clinical and hormonal evaluation. These diagnoses contributed to anovulation.

Almost 35% women were obese in our study and 22% overweight. A strong association between BMI and the occurrence of AUB. The most common pattern of AUB observed in our study was anovulatory bleeding (AUB-O), consistent with the hormonal and metabolic disturbances known to be associated with excess adiposity. Our findings match with the study by Shobeiri et al. [17] who reported a significant correlation between elevated BMI and the prevalence of AUB, especially anovulatory types. Also, Vural et al. [18] suggested that menstrual irregularities, including heavy regular periods and infrequent periods, were significantly more common in obese adolescents, emphasizing early metabolic influence on menstrual function. Anovulatory bleeding underscores the need for metabolic assessment in the workup of AUB, especially in overweight and obese women. Furthermore, these women may be at increased risk of endometrial hyperplasia or malignancy due to chronic unopposed estrogen exposure, which is supported by Wang and Akalyaa et al., reinforcing the importance of early detection and appropriate endometrial evaluation [17,19].

About two thirds were parous women. This has also been seen in several other studies, and it is associated with increased risk of AUB. This might be due to increasing age and changes due to pregnancy. A study by Singh et al. showed multiparous women were more likely to present with structural causes of AUB such as leiomyoma (fibroids) and adenomyosis, which were significantly associated with heavy bleeding [20].

On the other hand, some studies indicate that nulliparous, especially younger or unmarried women, may experience AUB due to anovulatory cycles and endocrine imbalances, such as polycystic ovarian syndrome (PCOS), rather than structural pathology [21]. Understanding the parity profile of women with AUB can help tailor investigations, for example, structural evaluation (e.g., ultrasound) may be prioritized in multiparous women, while endocrine workup may be recommended in nulliparous with irregular cycles.

Iron supplementation played a critical role in post-transfusion anemia management. Intravenous iron was administered in 30% of patients, while oral iron was given to 36.47%. These results reflect current clinical practice, where IV iron is increasingly favored for patients with moderate to severe iron deficiency anemia, particularly those with poor oral tolerance or ongoing bleeding [22]. Medical treatment is the first especially, non-hormonal treatment. This is consistent with global trends where NSAIDs and tranexamic acid are often the initial choices for women with contraindications to hormonal therapy or those desiring fertility preservation [23]. Oral progestogens were prescribed in 29% of cases, aligning with their known efficacy in managing heavy menstrual bleeding, especially in women with ovulatory disorders [24].

Combined oral contraceptive pills were less commonly used (6%), likely due to cultural and individual preferences, which may also explain the low uptake (12.4%) of the levonorgestrel-releasing intrauterine system (LNG-IUS or Mirena). The limited use of Mirena in our cohort reflects cultural resistance observed in some Middle Eastern populations, as reported in earlier studies examining contraceptive preferences and perceptions [25].

Some women had more than one structural abnormality, reinforcing the multifactorial etiology of AUB. These results are in line with previous studies identifying fibroids and adenomyosis as leading causes of AUB in premenopausal women [26,27]. Only a small number of women with malignancies (1.8%) were identified, aligning with literature indicating a low prevalence of cancer in women with AUB, particularly those without risk factors [28]. There was one case of leiomyosarcoma, two cases of endometrial cancer, and two cases of cervical cancer. These were referred promptly to gynecologic oncology. The low rate of malignancy (approximately 4%) is in line with existing literature emphasizing the need for selective and appropriate investigation in high-risk groups [29].

Endometrial hyperplasia was observed in 7% of cases, underlining the importance of biopsy in AUB management, particularly in women over 40 or those with risk factors. Not all of them had hysterectomy and some received MIRENA, a progestogen intrauterine device for treatment.

Surgical intervention was done in 42% of patients, with myomectomy (30.35%) being the most frequent procedure. This is comparable to international data where myomectomy remains a key fertility-sparing treatment in women with symptomatic fibroids [25].

Hysterectomy was performed in 15.78% of patients, typically in those with completed families or refractory symptoms. Minimally invasive procedures like hysteroscopic polypectomy (8.64%) and endometrial ablation (2.25%) were utilized based on patient suitability and pathology findings.

At short-term follow-up, most patients transitioned to moderate anemia with symptomatic relief, demonstrating that transfusion can provide effective acute management. However, recurrence of anemia in 3% of patients and the need for repeat transfusions in 23.67% underscore the chronic and recurrent nature of AUB in some individuals. Similar recurrence rates are reported in longitudinal studies of women with untreated or inadequately managed structural causes of AUB [26].

## 6. Conclusions

This study reinforces the fact that AUB is an important cause of severe anemia requiring blood transfusion. It highlights the need for a combined strategy of transfusion to alleviate acute symptoms, iron replacement to build up iron stores, and definitive treatment of the underlying etiology of the AUB to ensure sustainable improvement and prevent recurrence. The strong association between high BMI and anovulatory AUB demonstrated in this study suggests that weight management may be a key component in the prevention and treatment of menstrual irregularities. Prospective studies are needed to assess whether weight reduction and insulin-sensitizing therapies can restore ovulation and reduce blood transfusions in women with AUB.

The key limitations of the study are its retrospective nature and lack of long-term follow-up.

## Figures and Tables

**Table 1:** Baseline characteristics of the participants.

Parameters	Minimum	Maximum	Mean	SD
Age (years)	11	56	37.66	11.40
BMI (kg/m^2^)	13	78	29.40	8.90
Parity	0	12	3.13	3.20
Pulse BPM	60	149	90.08	16.40
Systolic BP mm Hg	70	179	114.40	19.61
Diastolic BP mm Hg	40	105	69.40	11.25
Shock index	0.51	1.41	0.829	1.02
Age of menarche (years)	9	17	12.70	1.46
Duration of bleeding (days)	3	60	8.31	4.37
Lowest Hb gms/dL at presentation	1.7	7.9	5.70	1.149
Serum ferritin (ng)	1	54	11.01	21.88
Transferrin saturation	1.7	87	7.36	10.34
No. of packed red cells units	1	10	2.38	1.20
Post blood transfusion Hb	5.2	11.2	8.36	0.97
Hb 3 month	4	15	10.20	1.77

**Table 2:** Correlation between the severity of anemia and units of blood transfusion.

Hb	Number of Units		*p*-Value
	Median	IQR	
Moderate (7.0–9.9)	1.50	1.00–2.00	<0.001 *
Severe (4.0–6.9)	2.00	2.00–3.00	
Very severe (<4.0)		3.00–4.00	
**Hb**	**Subsequent transfusion**		***p*-value**
	**No**	**Yes**	
	**n (%)**	**n (%)**	
Moderate (7.0–9.9)	22 (78.6)	6 (21.4)	0.044 ^†^
Severe (4.0–6.9)	161 (74.2)	56 (25.8)	
Very severe (<4.0)	20 (95.2)	1 (4.8)	

* Statistically significant; test: Kruskal–Wallis H. ^†^ Statistically significant; test: likelihood ratio.

**Table 3:** Etiology as per the FIGO-2 (PALM-COEIN) classification.

Causes as per FIGO	No. of Case	%
P	9	3.38
A	27	10.15
L	100	37.59
M	7	2.63
C	4	1.50
O	52	19.55
E	22	8.27
I	1	0.38
N	11	4.14
Unknown	1	0.38
AL	10	3.76
AE	1	0.38
AO	1	0.38
AP	1	0.38
EL	2	0.75
EO	4	1.50
LM	1	0.38
LO	3	1.13
LP	2	0.75
PO	2	0.75
CO	4	1.50
CA	1	0.38
Total	266	100.0

P—Polyp; A—Adenomyosis; L—Leiomyoma; M—Malignancy; C—Coagulation defects; E—Endometrial; O—Ovulatory; I—Iatrogenic. N—Not yet determined.

**Table 4:** Histopathological findings.

Polyp	23	20.53%
Leiomyoma and adenomyoma	16	14.29%
Leiomyoma	36	32.14%
Adenomyosis	24	21.43%
Leiomyosarcoma	1	0.89%
Endometrial hyperplasia	8	7.14%
Endometrial carcinoma	2	1.79%
Cervical cancer	2	1.79%

**Table 5:** Medical treatment used by the women.

Medical Treatment	No. of Cases	Percentage
Mirena intrauterine system	33	12.4
GnRH (gonadotropin releasing hormone)	15	5.64
Medroxyprogesterone	10	3.75
Norethisterone	62	23.34
Dydrogesterone	6	2.25
Yasmin combined pills	16	6.01
Mefenamic acid and tranexamic acid	217	81.5

## Data Availability

Data is available with Sunita Jesrani and asha Santhosh. Will be provided if needed.

## Supplementary Materials

The following supporting information can be downloaded at: https://www.mdpi.com/article/10.3390/life16050855/s1, Figure S1: STROBE Statement—checklist of items that should be included in reports of observational studies; Table S1: Strobe check list.
